# Effects of Dapagliflozin Adjunct to Insulin on Glycemic Variations in Patients with Newly Diagnosed Type 2 Diabetes: A Randomized, Controlled, Open-Labeled Trial

**DOI:** 10.1155/2021/6618257

**Published:** 2021-08-27

**Authors:** Lan-lan Jiang, Peng Zhang, Bing-li Liu, Reng-na Yan, Lei Ye, Jian-hua Ma, Feng-fei Li

**Affiliations:** ^1^Department of Endocrinology, Nanjing First Hospital, Nanjing Medical University, Nanjing, China; ^2^National Heart Research Institute Singapore, National Heart Centre Singapore, Singapore

## Abstract

**Background:**

This study is aimed at investigating whether dapagliflozin adjunct to insulin therapy further improves glycemic control compared to insulin therapy alone in patients with newly diagnosed type 2 diabetes (T2D).

**Methods:**

This single-centre, randomized, controlled, open-labeled trial recruited newly diagnosed T2D patients. Subjects were randomized 1 : 1 to the dapagliflozin add-on to continuous subcutaneous insulin infusion (CSII) group (DAPA) or the CSII therapy group for 5 weeks. Standard meal tests were performed 3 times at days -3, 7, and 35 for glucose, C-peptide, and insulin level determination. Two-time continuous glucose monitoring (CGM) was performed at baseline and at the end of the study. The primary endpoint was the difference in the mean amplitude of glycemic excursions (MAGEs) between the groups.

**Results:**

A total of 66 subjects completed the study, with 34 and 32 patients in the DAPA and CSII groups, respectively. Patients in the DAPA group exhibited significant decreases in MAGE levels at the endpoint. We also observed that patients in the DAPA group had a lower homoeostasis model assessment insulin resistance (HOMA-IR) and a higher homoeostasis model assessment B (HOMA-B) value at 1 week and 5 weeks compared to those with insulin therapy, respectively. In addition, our data showed that patients in the DAPA group showed a significantly lower insulin dose (0.07 U/kg) and weighed less than those in the CSII group.

**Conclusion:**

Our data indicate that dapagliflozin adjunct to insulin is a safe and effective therapy for improving glycemic variations, insulin sensitivity, and weight loss in newly diagnosed T2D patients.

## 1. Introduction

The incidence and prevalence of type 2 diabetes (T2D) are increasing worldwide partly due to changes in dietary habits and a lack of exercise [1–3]. Insulin therapy is the mainstay of treatment for achieving target hemoglobin A1c (HbA_1c_) levels in patients with T2D. However, this treatment is associated with hypoglycemia [4–6] (especially in older male diabetic patients [7]), increased blood pressure [8], and weight gain [9]. Other concerns regarding the use of insulin, such as adherence, preferences, and resource allocation, have also been well documented [10]. Moreover, intensification of insulin is associated with glucagon imbalances and increased rate of gastric emptying, which may lead to a dramatically increased glycemic variations (GV) [11]. As such, an adjunct agent for insulin to provide decreasing hypoglycemia, weight gain, blood pressure, and GV is necessarily needed.

Dapagliflozin, a member of sodium-glucose cotransporter-2 (SGLT2) inhibitors, was recently approved for the treatment of T2D. Dapagliflozin brings to improvement in glucose control mainly by targeting the pathophysiologic increase in renal glucose reabsorption, leading to increased glucose excretion in the urine [12]. The pleiotropic effects beyond glucose-lowering in T2D associated with macro- and microvascular complications have been proven [13]. Of importance, previous studies have demonstrated that patients treated with dapagliflozin have significant improvements in glycemic control [14–19], with no increase in hypoglycemia [20]. We have previously reported that newly diagnosed T2D patients treated with dapagliflozin alone for 24 weeks show improvement in GV and a reduction in oxidative stress, compared to those receiving placebo treatment [21]. Therefore, dapagliflozin should be considered as a good candidate adjunct to insulin to avoid dramatic changes of GV in patients with T2D. However, the effects of dapagliflozin adjunct to insulin on GV in T2D patients remain elucidated.

Therefore, we aimed to observe whether dapagliflozin adjunct to insulin therapy further improved glycemic control in patients with newly diagnosed T2D compared to insulin therapy alone.

## 2. Methods and Materials

This was a single-centre, randomized, controlled, open-labeled trial performed at the Department of Endocrinology, Nanjing First Hospital, Nanjing Medical University, between July 2018 and March 2019. The inclusion criteria were as follows: (1) patients aged between 18 and 75 years, (2) HbA_1c_ ≥ 9.0% at screening, and (3) stabilized weight for at least 12 weeks. Patients were excluded from the analysis if they had acute diabetic complications, chronic liver disease (ALT > 2 times upper limit of the reference value), kidney disease (Estimated Glomerular Filtration Rate (eGFR) < 60 ml/(min∗1.73m^2^), eGFR calculated by the MDRD Study Equation), an infection condition, or cardiovascular disease.

All procedures followed were in accordance with the ethical standards of Nanjing First Hospital and with the Helsinki Declaration of 1964, as revised in 2013. Informed consent was obtained from all the patients for inclusion in the study.

**Figure 1:**
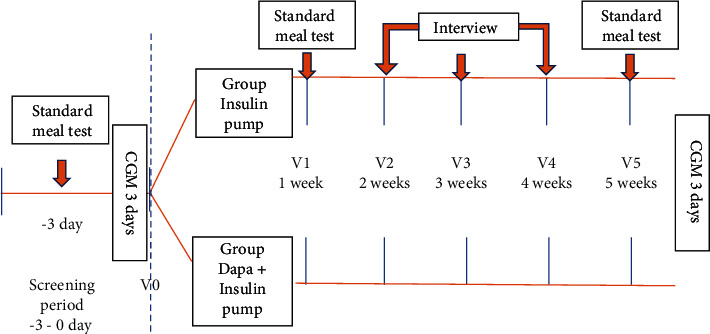
Study flow chart.

After baseline parameters were assessed, patients were randomly assigned in a 1 : 1 ratio to receive 10 mg dapagliflozin (Bristol-Myers Squibb, Lawrenceville, NJ), plus continuous subcutaneous insulin infusion (CSII), referred to as the DAPA group, or CSII therapy alone, CSII group, for 5 weeks (1 week in hospital and another 4 weeks at home). The total daily insulin (Aspart, Novo Nordisk, Bagsværd, Denmark) dose was 0.5 IU/kg which was given in two injection modes: half the total daily dose was equally given as boluses with three meals, while the remaining insulin was given as a basal dose. Investigators titrated insulin doses on an individual-patient basis using the titration algorithm, as described previously [22]. Scheduled visits occurred once a week for the 5 weeks. The treatment protocol remained unchanged during the study period (Figure 1). Patients were recharged if they achieved 80% fasting blood glucose levels within 4.4-7.0 mmol/L and postprandial glucose within 4.4-10.0 mmol/L for 3 consecutive days. Patients were instructed to maintain their usual diet and exercise at home, and investigations titrated insulin doses according to -2 days of self-monitoring blood glucose (before and 2 h after three meals each day and before bedtime) on each scheduled weekly visit. Investigators titrated insulin doses on an individual-patient basis using the titration algorithm: if the fasting blood glucose level was less than 4.4 mmol/L, the basal insulin dose was reduced by 2 units; if the fasting blood glucose level was within 4.4-7.0 mmol/L, the basal insulin dose was unchanged; if the fasting blood glucose level was within 6.2-7.8, 7.9-10.0, or >10.0 mmol/L, the basal insulin dose was increased subsequently by 2, 4, or 6 units, respectively; if the postprandial blood glucose level was less than 4.4 mmol/L, the bolus insulin dose was reduced by 2 units; if the postprandial blood glucose level was within 4.4-10.0 mmol/L, the bolus insulin dose was unchanged; if the postprandial blood glucose level was >10.0 mmol/L, the bolus insulin dose was increased subsequently by 2 units, respectively, as described previously [23, 24].

All recruited patients were subjected to two-time continuous glucose monitoring (CGM) (Sof-sensor, CGMS-Gold, Medtronic Incorporated, Northridge, USA) for days -3-0 and 36-39 of the study. The CGM sensor was subcutaneously embedded at day -3 and day 36 at 16 : 00-17 : 00 PM. During the two-time 3-day CGM periods, the investigator nurses checked the sensor and entered at least 4 calibration readings each day. The sensors were removed, and the CGM data were saved by the investigator, as described previously [22, 25, 26]. All patients were served three meals at 0700, 1100, and 1700, consisting of a total daily caloric intake of 25 kcal/kg/day. The proportions of carbohydrates, proteins, and fats were 55%, 17%, and 28%, respectively. In addition, standard meal tests were performed on days -3, 7, and 35. Serum samples were collected at 0, 30, and 120 min after meals for determination of glucose, insulin, and C-peptide concentrations.

The 24 h mean glucose (MG), the standard deviation of the MG (SDMG), the mean amplitude of glycemic excursions (MAGEs), the incremental area under curve (AUC) of blood glucose above 10.0 mmol/L, the incremental area over the curve (AOC) less than 3.9 mmol/L, and the time in target range (TIR) were recorded and calculated, as previously described [22, 26]. Plasma insulin levels were determined using an insulin radioimmunoassay kit (Beijing Technology Company, Beijing, China). HbA_1c_ was measured by a DiaSTAT HbA_1c_ analyzer (Bio-Rad, Hercules, CA, USA). C-peptide and glucose concentrations were measured centrally at the central laboratory in Nanjing First Hospital, Nanjing Medical University. Beta cell function was assessed by the homoeostasis model assessment B (HOMA-B), and insulin sensitivity was indicated by HOMA-IR [27, 28].

The primary endpoint was the difference in MAGE between the two groups at the endpoint. Secondary outcomes were the differences in precise insulin doses, hourly MG, 24 h MG, SDMG, CV, incremental AUC of hyperglycemia, incremental AOC of hypoglycemia, and weight change from baseline to the completion of treatments. Beta cell function was assessed by HOMA-B, and the insulin sensitivity (HOMA-IR) was also analyzed.

This study was registered with ClinicalTrials.gov (NCT04120623).

### 2.1. Statistical Analysis

Data were analyzed using the SPSS PASW Statistics 18 Package. The Shapiro-Wilk test was used to assess the distribution of data. Normally distributed and continuous variables were presented as mean ± standard deviation (SD), while nonnormally distributed variables were presented as median (interquartile range). An independent *t*-test and Wilcoxon test were used in the comparisons between groups. The mixed ANOVA model (2 × 2) test was used to compare differences between groups. A Bonferroni correction was also performed. *P* values were two-tailed with a significance level of 5%.

## 3. Results

### 3.1. Baseline Characteristics

A total of 66 newly diagnosed T2D patients who met the inclusion criteria were admitted to the study, 34 in the DAPA group and 32 in the CSII group. All recruited subjects completed the study (Table 1). Importantly, there were no significant differences in the demographic characteristics of the patients between the two groups.

### 3.2. Glycemic Variations

**Figure 2:**
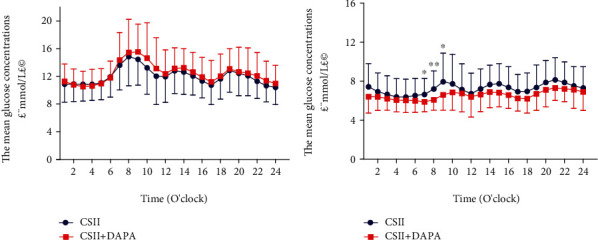
Hourly glucose concentrations between the two groups: (a) before therapy and (b) after therapy. Red line: CSII+DAPA group and blue line: CSII group; ^∗^*P* < 0.05; ^∗∗^*P* < 0.01.

Subjects in the DAPA group exhibited significant decreases in the MAGE, incremental AUC hyperglycemia, incremental AOC of hypoglycemia, and TIR at the endpoint of the study compared to those in the CSII group (Table 2). We also observed a trend towards a reduction in the SDMG, 24 h MG, and the CV in patients in the DAPA group compared to the CSII group; however, these were not significant. We calculated readings delivered from CGM every 5 min, and we observed that patients in the DAPA group had a significant decrease in hourly mean glucose at 0700, 0800, and 0900 compared to those in the CSII group (Figure 2). As patients had their meals at 0700, 1100, and 1700, we calculated the incremental AUC of glucose before and after each meal. Our data showed that patients in the DAPA group had a statistically significant decrease in AUC 1 h before breakfast and 1 h, 2 h, and 3 h after breakfast (Table 3). Hypoglycemia is an important concern regarding insulin therapy in patients with T2D. Therefore, we identified hypoglycemia episodes from CGM readings and found that none of the patients in the DAPA group experienced hypoglycemia. However, a total of 4 patients had hypoglycemic episodes in the CSII group (*P* = 0.05) (Table 2).

### 3.3. Beta Cell Function and Insulin Sensitivity

To determine the effect of dapagliflozin therapy on beta cell function and insulin sensitivity in patients with newly diagnosed T2D, we compared HOMA-B and HOMA-IR at 1 week of treatment and the endpoint after treatment completion between the two groups. Our data showed that, as expected, patients in the DAPA group had lower HOMA-IR values than those in the CSII group at 1 week of this study, and this improvement remained at the endpoint (*P* < 0.05, for both). Interestingly, patients in the DAPA group did not show significant improvement in HOMA-B at 1 week (*P* = 0.06), but achieved a significantly increase in HOMA-B values after 5 weeks of dapagliflozin adjunct to insulin therapy compared to those in the CSII group (*P* = 0.01) (Table 4).

### 3.4. Insulin and Weight Gain

Patients in the DAPA group reached glycemic goals in a shorter amount of time than those in the CSII group (3.07 ± 0.92 days vs. 3.89 ± 1.14 days, *P* < 0.01). The daily total insulin dose required by subjects to maintain euglycemic control in the DAPA group was significantly lower than that of the CSII group after 5 weeks (0.23 ± 0.09 U/kg vs. 0.30 ± 0.11 U/kg, *P* = 0.02). We next compared the bolus insulin and basal insulin doses required in the patients between the two groups. We observed that patients in the DAPA group required significantly lower bolus insulin doses compared to those in the CSII group (*P* = 0.05), and our data also indicated that dapagliflozin treatment exhibited a significantly lower basal insulin doses after 5 weeks (*P* = 0.03) (Table 5). In addition, we observed that patients receiving dapagliflozin treatment had a significant reduction in body weight compared to those receiving insulin therapy (*P* < 0.01) (Table 1).

In addition, we also observed a remission of 52.9% and 46.9% in patients with CSII+DAPA and CSII alone therapy after one-year follow-up, respectively.

### 3.5. Safety and Tolerance

We compared the experienced side effects during the study period. One patient experienced a moderate urinary tract infection during dapagliflozin therapy at 2 weeks, but he continued to complete the study. Other patients tolerated the dapagliflozin or insulin therapy well, without any adverse reactions recorded.

## 4. Discussion

We conducted a prospective study on patients with newly diagnosed T2D and demonstrated that dapagliflozin add-on intensive insulin therapy led to a significant improvement in GV. We also observed that patients receiving dapagliflozin with insulin had improved insulin sensitivity and beta cell function.

Studies have demonstrated that dapagliflozin can be well tolerated in patients with T2D for more than 2 years [20, 29], accompanying with blood pressure reduction and cardiovascular and renal benefits 13. Importantly, clinical trials have suggested the potential benefits of SGLT2 inhibitors as an adjunctive treatment for type 1 diabetes (T1D) [30], especially in combination with insulin therapy to improve glycemic control in patients with inadequately controlled TID [31]. The DEPICT-2 Study demonstrated that dapagliflozin at 5 mg or 10 mg add-on insulin therapy was safe and well tolerated and exhibited a potential benefit in improving glycemic control and hypoglycemia in T1D [32]. In our study, type 2 diabetic patients received dapagliflozin (10 mg) adjunctive to insulin therapy were well tolerated for 5 weeks.

As expected, our CGM data showed that patients treated with dapagliflozin exhibited significantly improved GV, such as MAGE, incremental AUC of hyperglycemia, and TIR. Importantly, patients who received dapagliflozin had a statistically significant decrease in the incremental AOC of hypoglycemia. Our data were consistent with studies reporting that dapagliflozin had the ability to improve glycemic control [14–19], with no increase in hypoglycemia [20]. Furthermore, we observed that patients who received dapagliflozin in combination with insulin therapy showed a reduction in 24 h MBG, which was consistent with our previous study showing that subjects with dapagliflozin therapy had significantly reduced 24 h MBG compared with placebo after 24 weeks of treatment [21]. Dapagliflozin reduces hyperglycemia, body weight, and systolic blood pressure [17, 33] and increases atrial natriuretic peptide levels [34], which may contribute to the decrease in the incidence of cardiovascular disease (CVD). The reduction we showed in glycemic variations may contribute to the reduction of decrease in CVD risk, as acute glucose variations during postprandial periods had a potential role in oxidative stress in patients with T2D [35]. Furthermore, a large GV induced the overproduction of peroxynitrite and nitrotyrosine, which impaired endothelial cell functions [35, 36].

Dapagliflozin add-on to insulin therapy leads to a reduction in insulin dose and weight loss, without any increases in hypoglycemia in T1D [37]. In this study, we observed that dapagliflozin as an adjunct to insulin significantly reduced basal and bolus insulin doses in T2D after 5-week treatment, with no weight changes and hypoglycemia. We also analyzed *β*-cell function and insulin sensitivity in patients between groups. Interestingly, subjects receiving dapagliflozin add-on therapy had statistically improved in insulin sensitivity and beta cell function. However, our data indicated that patients achieved the recovery of beta cell function was seen at the endpoint (5-week treatment), which may be the reason of SGLT2 inhibitors have the potential ability to preserve beta cell mass in diabetic mouse model [38].

An array of metrics delivered from CGM could be used to interpret the GV in T1D and T2D [39–41]. Our CGM data showed that patients receiving dapagliflozin in combination with insulin therapy showed a significant improvement in MAGE, incremental AUC, and TIR. In addition, we also observed that subjects in the DAPA group had a significant decrease in the incremental AUC at 3 h postbreakfast compared to the CSII group. Therefore, it would be more logical to hypothesis that patients with higher HbA_1c_ values may harvest more benefit from dapagliflozin adjunct to insulin therapy for the improvement in incremental AUC after breakfast. However, our data could not address the underlying mechanisms of this decrease in incremental AUC after breakfast. We could infer that decreased incremental AUC after breakfast might be the reason for nearly half of the newly diagnosed T2D patients with only abnormal postprandial glucose concentrations [42]. Furthermore, isolated postprandial hyperglycemia is more prominent in Chinese patients compared to Western patients [42, 43]. This was strengthened by our study reporting that newly diagnosed T2D patients with higher HbA_1c_ values had larger GV and higher peak glucose concentrations after breakfast. Limitations of this study should be addressed; in particular, the study population was relatively small, and the observation time was relatively short. However, the strength and novelty of this study should also be addressed, especially this study was performed under very controlled conditions in the hospital setting (which is quite unique) and with the use of CGM, thus providing valuable and reliable data on the effect of dapagliflozin on GV in patients managed with state-of-the-art CSII.

In conclusion, our data indicate that dapagliflozin adjunct to insulin is a safe and effective therapy to improve glycemic variations, insulin sensitivity, and weight loss in newly diagnosed T2D patients.

## Figures and Tables

**Table 1:** Baseline characteristics of patients.

Parameter	Before therapy			After therapy (5 w)				
	DAPA+CSII (*n* = 34)	CSII (*n* = 32)	*P* value	DAPA+CSII (*n* = 34)	CSII (*n* = 32)	△DAPA+CSII	△CSII	*P* value
Gender (M/F)	34 (22/12)	32 (26/6)	0.13	/	/	/	/	/
Age (years)	50.0 ± 10.4	46.3 ± 10.1	0.15	/	/	/	/	/
Weight (kg)	72.6 ± 9.9	69.5 ± 12.2	0.26	70.0 ± 9.7	68.2 ± 12.1	−2.6 ± 0.7	−1.3 ± 0.8	<0.01^∗∗^
BMI (kg/m^2^)	25.6 ± 3.0	24.1 ± 3.4	0.08	24.7 ± 3.0	23.7 ± 3.4	−0.9 ± 0.2	−0.45 ± 0.3	<0.01^∗∗^
SBP (mmHg)	125.9 ± 13.3	125.4 ± 12.0	0.89	120.5 ± 8.6	120.5 ± 10.6	−5.4 ± 13.0	−4.9 ± 14.1	0.90
DBP (mmHg)	81.6 ± 7.5	81.7 ± 4.8	0.97	78.7 ± 5.9	80.9 ± 4.6	−2.6 ± 5.1	−0.7 ± 4.8	0.09
Na^+^ (mmol/L)	142.6 ± 2.4	142.8 ± 2.8	0.74	143.9 ± 2.2	143.0 ± 2.5	−1.5 ± 3.0	−0.2 ± 3.3	0.14
HbA_1c_ (%)	10.2 ± 1.77	10.5 ± 1.6	0.52	7.8 ± 0.9	8.1 ± 1.0	−2.5 ± 1.2	−2.4 ± 1.2	0.93
FBG (mmol/L)	11.9 ± 2.8	11.3 ± 2.0	0.30	6.9 ± 1.4	7.5 ± 2.0	−5.0 ± 3.1	−3.8 ± 2.3	0.08

BMI: body mass index; SBP: systolic blood pressure; DBP: diastolic blood pressure; FBG: fasting blood glucose; HbA_1c_: hemoglobin A1c. Data were presented as means ± SD; ^∗^*P* < 0.05; ^∗∗^*P* < 0.01. △: before therapy-after therapy (5 w).

**Table 2:** Blood glucose variability in the recruited subjects.

Parameter	Before therapy		*P* value	After therapy (5 w)		*P* value
	DAPA+CSII	CSII		DAPA+CSII	CSII	
MAGE	6.25 ± 2.55	5.97 ± 2.47	0.68	2.34 ± 1.10	3.46 ± 2.33	0.03^∗^
MBG	12.15 ± 2.37	12.28 ± 2.14	0.83	6.60 ± 0.98	7.19 ± 1.65	0.10
SD	2.52 ± 0.99	2.50 ± 0.90	0.94	1.09 ± 0.63	1.42 ± 0.70	0.07
CV%	20.54 ± 6.48	20.20 ± 5.95	0.84	16.21 ± 7.90	19.27 ± 7.79	0.14
AUC > 10 mmol/L	2.05 (1.19, 4.20)	2.48 (0.90, 3.93)	0.89	0 (0, 0)	0.02 (0, 0.40)	0.01^∗^
AOC < 3.9 mmol/L	0 (0, 0)	0 (0, 0)	0.33	0 (0, 0)	0 (0, 0)	0.03^∗^
Hypoglycemia (*n*)	0	0	/	0	4	0.05
TIR	26.35 ± 26.64	31.95 ± 25.17	0.42	96.52 ± 8.28	85.09 ± 19.08	0.01^∗^

MAGE: the mean amplitude of glycemic excursion; MBG: 24 h mean blood glucose; SD: standard deviation of mean glucose; CV%: the coefficient of variation; AUC > 10 mmol/L: the incremental area under the curve (AUC) of a glucose level > 10.0 mmol/L; AOC < 3.9 mmol/L: the incremental area over the curve (AOC) of a glucose level < 3.9 mmol/L; TIR: the time in target range (3.9-10.0 mmol/L). Data were presented as means ± SD or IQR. ^∗^*P* < 0.05; ^∗∗^*P* < 0.01.

**Table 3:** The incremental AUC of glucose values before and after each meal.

Parameter	Before therapy		*P* value	After therapy		*P* value
	DAPA+CSII	CSII		DAPA+CSII	CSII	
Breakfast						
AUCb-1 h	3.04 ± 0.86	2.86 ± 0.76	0.42	1.22 ± 0.21	1.38 ± 0.34	0.03^∗^
AUCa-1 h	3.22 ± 0.99	3.10 ± 0.87	0.62	1.27 ± 0.23	1.51 ± 0.40	0.01^∗^
AUCa-2 h	6.45 ± 1.73	6.10 ± 1.59	0.42	2.64 ± 0.52	3.17 ± 0.98	0.01^∗^
AUCa-3 h	9.49 ± 2.67	8.85 ± 2.20	0.32	4.08 ± 0.78	4.77 ± 1.54	0.03^∗^
AUCa-4 h	12.21 ± 3.51	11.34 ± 2.83	0.31	5.48 ± 1.01	6.25 ± 1.98	0.06

Lunch						
AUCb-1 h	2.72 ± 0.92	2.50 ± 0.85	0.34	1.40 ± 0.40	1.48 ± 0.55	0.53
AUCa-1 h	2.58 ± 0.70	2.50 ± 0.76	0.67	1.33 ± 0.43	1.40 ± 0.43	0.58
AUCa-2 h	5.32 ± 1.26	5.17 ± 1.36	0.65	2.72 ± 0.76	2.91 ± 0.83	0.37
AUCa-3 h	8.08 ± 1.74	7.80 ± 1.93	0.56	4.15 ± 1.00	4.51 ± 1.13	0.21
AUCa-4 h	10.70 ± 2.26	10.30 ± 2.41	0.52	5.58 ± 1.19	6.12 ± 1.48	0.13

Dinner						
AUCb-1 h	2.33 ± 0.65	2.23 ± 0.59	0.54	1.30 ± 0.27	1.44 ± 0.36	0.10
AUCa-1 h	2.52 ± 0.67	2.45 ± 0.61	0.66	1.28 ± 0.34	1.45 ± 0.40	0.08
AUCa-2 h	5.24 ± 1.16	5.12 ± 1.21	0.70	2.64 ± 0.73	3.00 ± 0.84	0.09
AUCa-3 h	7.87 ± 1.82	7.70 ± 1.79	0.72	4.08 ± 1.13	4.64 ± 1.26	0.08
AUCa-4 h	10.46 ± 2.44	10.21 ± 2.28	0.69	5.69 ± 1.13	6.34 ± 1.62	0.08

AUC: area under the curve (day∗mmol/L); AUCb: area under the curve before meal (day∗mmol/L); AUCa: area under the curve after meal (day∗mmol/L); ^∗^*P* < 0.05.

**Table 4:** Beta cell function and insulin sensitivity profiles between the two groups.

Parameter	Before therapy		*P* value	After therapy (1 w)				*P* value	After therapy (5 w)				*P* value
	DAPA+CSII	CSII		DAPA+CSII	CSII	△DAPA+CSII	△CSII		DAPA+CSII	CSII	△DAPA+CSII	△CSII	
GLU0 min	11.9 ± 2.8	11.3 ± 2.0	0.30	6.3 ± 1.4	6.9 ± 0.9	−5.6 ± 2.7	−4.5 ± 1.8	0.06	6.9 ± 1.4	7.5 ± 1.0	−5.0 ± 3.1	−3.8 ± 2.3	0.08
GLU30 min	14.7 ± 3.3	13.6 ± 3.6	0.20	9.0 ± 1.7	9.8 ± 1.3	−5.7 ± 3.1	−3.9 ± 3.1	0.02	8.9 ± 1.7	9.4 ± 2.6	−5.8 ± 3.5	−4.2 ± 3.8	0.09
GLU120 min	21.2 ± 3.9	19.2 ± 4.7	0.07	14.4 ± 3.0	15.2 ± 3.5	−6.7 ± 4.1	−4.0 ± 4.1	0.01	12.1 ± 2.4	12.4 ± 3.6	−9.1 ± 4.4	−6.7 ± 5.3	0.02
INS0 min	10.0 ± 5.5	7.1 ± 3.5	0.02	5.8 ± 3.8	4.4 ± 2.1	-4.0 (-6.78, -0.6)	-2.2 (-4.2, 0.1)	0.11	8.7 ± 4.1	6.3 ± 3.1	-0.8 (-3.2, 1.6)	-0.5 (-1.9, 0.8)	0.70
INS30 min	15.4 ± 9.5	10.7 ± 4.7	0.02	13.9 ± 9.6	10.13 ± 5.7	-1.4 (-5.0, 2.3)	-1.3 (-3.9, 2.4)	0.71	16.2 ± 8.6	13.6 ± 10.8	1.5 (-3.6, 5.4)	-0.9 (-4.1, 8.6)	0.10
INS120 min	23.9 ± 13.8	17.9 ± 11.6	0.07	32.3 ± 29.6	18.6 ± 11.9	3.2 (0.7, 9.5)	0.7 (-4.5, 7.5)	0.19	33.7 ± 29.7	22.3 ± 14.2	6.0 (-5.4, 14.6)	1.8 (-2.6, 12.7)	0.55
CP0 min	2.5 ± 0.9	1.9 ± 0.7	0.01	1.9 ± 0.8	1.6 ± 0.5	-0.5 (-1.2, -0.2)	-0.2 (-0.7, -0.1)	0.06	1.9 ± 0.8	1.6 ± 0.6	-0.5 (-0.9, -0.1)	-0.4 (-0.8, -0.1)	0.41
CP30 min	3.0 ± 1.1	2.3 ± 0.7	0.01	2.6 ± 1.1	2.1 ± 0.8	-0.2 (-0.9, 0.2)	-0.1 (-0.7, 0.2)	0.52	2.6 ± 1.0	2.4 ± 1.3	-0.3 (1.0, 0.3)	-0.4 (-0.8, 0.7)	0.42
CP120 min	4.3 ± 1.7	3.5 ± 1.4	0.04	5.6 ± 2.6	4.0 ± 1.7	1.0 (0.1, 2.1)	0.3 (-0.6, 1.7)	0.12	5.6 ± 3.3	4.5 ± 2.0	0.6 (-0.2, 2.0)	0.4 (-0.6, 2.6)	0.45
GLUCA0 min	128.5 ± 28.3	125.1 ± 23.2	0.60	126.9 ± 30.9	118.5 ± 34.9	-5.8 (24.5, 22.7)	-8.5 (-28.2, 3.1)	0.47	161.5 ± 42.8	145.9 ± 27.7	29.5 (-4.7, 69.4)	21.3 (-0.8, 50.9)	0.36
GLUCA30 min	137.6 ± 32.8	129.2 ± 25.3	0.25	128.8 ± 24.1	124.9 ± 27.0	-5.0 (-38.1, 17.3)	-2.2 (-27.1, 18.2)	0.64	165.0 ± 37.5	150.5 ± 30.7	17.9 (-12.9, 74.6)	24.6 (-8.0, 51.9)	0.75
GLUCA120 min	134.8 ± 26.8	125.7 ± 22.4	0.14	128.6 ± 21.7	128.6 ± 37.0	-3.7 (-21.8, 7.6)	2.9 (-21.5, 18.7)	0.34	161.5 ± 41.8	145.3 ± 38.2	12.5 (-7.5, 61.6)	27.3 (-3.5, 50.5)	0.93
HOMA-IR	5.0 (3.4, 12.1)	3.2 (2.0, 4.5)	0.08	1.2 (0.9, 1.9)	1.4 (0.9, 1.8)	-3.1 (-4.7, -1.8)	-1.9 (-2.8, -1.0)	0.01	2.4 (1.6, 3.3)	2.0 (1.2, 3.0)	-1.7 (-3.8, -1.1)	-1.0 (-2.0, -0.4)	0.03
HOMA-*β*	25.9 (12.5, 35.2)	20.1 (9.9, 28.6)	0.13	42.3 (22.9, 70.7)	27.2 (18.4, 37.2)	16.5 (2.4, 40.3)	6.4 (1.0, 15.6)	0.06	51.1 (29.3, 76.8)	33.7 (22.9, 39.7)	19.5 (12.9, 48.0)	8.7 (3.8, 22.7)	0.01

Glu0 min: glucose 0 min before the standard meal; Glu30 min: glucose 30 min after the standard meal; Glu120 min: glucose 120 min after the standard meal; INS0 min: insulin 0 min before the standard meal; INS30 min: insulin 30 min after the standard meal; INS120 min: insulin 120 min after the standard meal; CP0 min: C-peptide 0 min before the standard meal; CP30 min: C-peptide 30 min after the standard meal; CP120 min: C-peptide 120 min after the standard meal; GLUCA0 min: glucagon 0 min before the standard meal; GLUCA30 min: glucagon 30 min after the standard meal; GLUCA120 min: glucagon 120 min after the standard meal. HOMA-IR: homoeostasis model assessment insulin resistance; HOMA-B: homoeostasis model assessment B. △: before therapy-after therapy.

**Table 5:** Insulin doses required by patients in the two groups.

Parameter	Therapy for 1 week		*P* value	Therapy for 5 weeks		*P* value
	DAPA+CSII	CSII		DAPA+CSII	CSII	
Total (U/kg)	0.36 ± 0.12	0.39 ± 0.12	0.45	0.23 ± 0.09	0.30 ± 0.11	0.02
Basal (U/kg)	0.20 ± 0.07	0.21 ± 0.06	0.49	0.12 ± 0.06	0.16 ± 0.06	0.03
Total bolus (U/kg)	0.17 ± 0.07	0.18 ± 0.07	0.50	0.11 ± 0.05	0.14 ± 0.06	0.03
Bolus (B)	0.06 ± 0.03	0.06 ± 0.03	0.56	0.04 ± 0.02	0.05 ± 0.02	0.05
Bolus (L)	0.06 ± 0.02	0.06 ± 0.02	0.60	0.08 ± 0.02	0.05 ± 0.02	0.03
Bolus (D)	0.06 ± 0.02	0.06 ± 0.02	0.44	0.03 ± 0.01	0.04 ± .022	0.05

Bolus (B): bolus breakfast; bolus (L): bolus lunch; bolus (D): bolus dinner.

## Data Availability

The datasets generated and/or analyzed during the current study are not publicly available due to some reasons but are available from the corresponding author on reasonable request.
